# Torticollis as the Main Presentation in a Child with Russell-Silver Syndrome: A Case Report

**DOI:** 10.1155/2012/109416

**Published:** 2012-11-01

**Authors:** Mohsen Javadzadeh, Hedieh Saneifard, Amir Hossein Hosseini

**Affiliations:** ^1^Imam Hossein Hospital, Shahid Beheshti University of Medical Sciences, Tehran, Iran; ^2^Tajrish Hospital, Shahid Beheshti University of Medical Sciences, Tehran, Iran

## Abstract

Russell-Silver syndrome is a genetic disorder the inheritance pattern of which is mostly sporadic. Some of the features of the syndrome are present at birth, and others appear in later years. The main clinical features include low birth weight, poor growth postnatally, short height, and discrepancies in size between the two sides of the body Abu-Amera et al. (2008), Binder et al. (2011). There is no statistical significant difference in prevalence between males and females. We report a case of Russell-Silver syndrome with intrauterine and postnatal growth retardation, triangular face, and body asymmetry, in addition to torticollis as a novel manifestation. In neck sonography, we found asymmetry of sternocleidomastoid muscles. In conclusion, we describe torticollis as a presentation of Russell-Silver syndrome.

## 1. Introduction 

 Russell-Silver syndrome (RSS) was first described in 1953; it is a genetically heterogeneous imprinting syndrome that affects approximately 1/50000 children [1]. This syndrome is characterized by severe intrauterine growth retardation (IUGR) accompanied by postnatal growth deficiency. The birth weight of affected infants is typically two or more standard deviations (SDs) below the mean, and postnatal growth is two or more SD below the mean for length or height [1].

 Affected individuals typically have proportionately short stature, with normal head circumference. Some other features which are less common are fifth-finger clinodactyly, typical facial features with triangular faces characterized by broad forehead and narrow chin, and limb-length discrepancy that may result from hemihypertrophy with diminished growth of the affected side [2]. 

 Several helpful diagnostic scoring systems have been developed for RSS, and the more recent studies of Netchine et al. [3] and Bartholdi et al. [4] focused primarily on phenotypic findings. Nonetheless, many persons with RSS lack typical clinical features and have a more subtle presentation that is not consistent in all patients. For these atypical cases who lack the cardinal features of this syndrome and the clinical diagnosis that may not be made with certainty, genetic tests may be used to help to confirm the diagnosis in a significant percent of patients. Though the above tests help in confirming the diagnosis in doubtful cases, there are no tests to definitely rule out the diagnosis [3, 4].

 We describe a 10-year-old girl with all of the main clinical features of RSS. Along with the typical clinical findings, she had torticollis, which has not been described before in this syndrome even among the minor features, let alone as the presenting complaint.

## 2. Case

**Figure 1:**
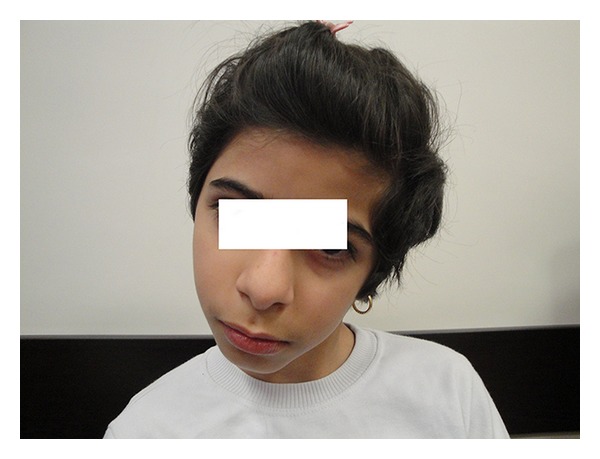


**Figure 2:**
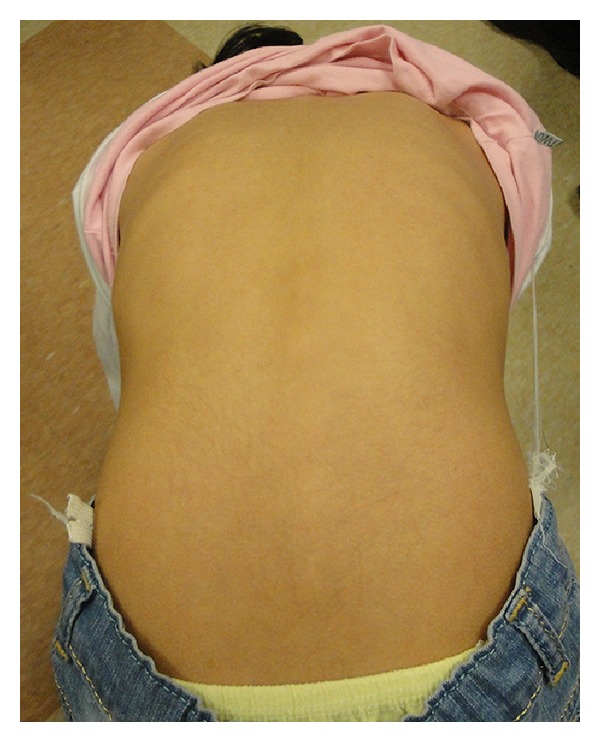


**Figure 3:**
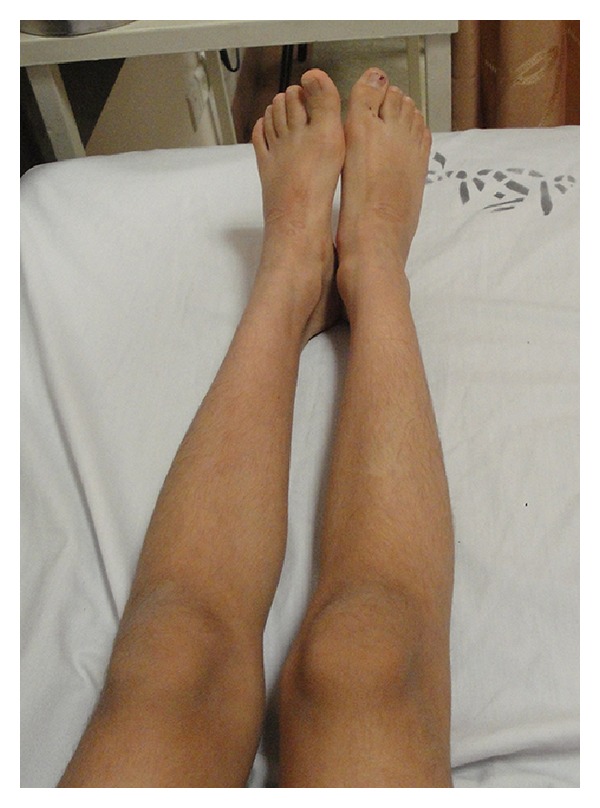


**Figure 4:**
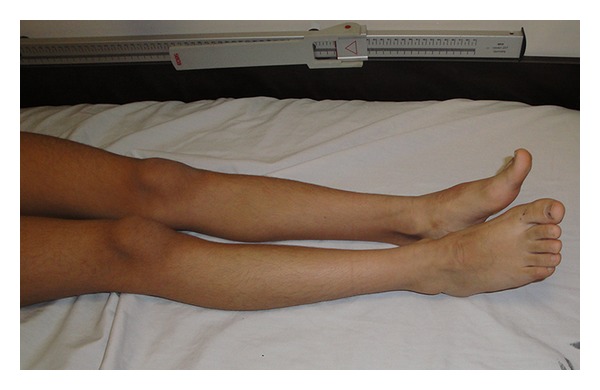


 A 10-year-old girl was admitted to our hospital with the chief complaints of progressive torticollis and limping. Parents had noticed the torticollis since a few weeks earlier, but they reported that limping had begun since 3 years earlier and had become worse throughout the years. Conception was natural and the pregnancy was uncomplicated. Patient was born on 39 weeks of gestation, by caesarian section. Fetal ultrasound was reported as normal in the first and the second trimesters. IUGR and oligohydramnios were detected only one week prior to birth. Birth weight was 2250 g (<10th weight for gestational age percentile). Apgar score was 8 at 5 and 9 at 10 minutes. There were no respiratory, neurologic, metabolic, or infectious complications at neonatal and infantile period. The patient and her parents denied the history of any drug consumption or abuse. She suffered from failure to thrive with sparing of her head circumference. Her neurological development had been within normal limits initially but the parents reported that their daughter had become less and less communicative gradually over the years. She had developed a slurred speech and recently had much difficulty in performing the school tasks. She also suffered from limping which the parents thought it to be because of right lower limb weakness. Due to poor control over right hand, especially during writing, she had become an obligatorily left handed. Two weeks before her admission in our hospital, she developed an acute-onset, nontraumatic left-sided neck deviation. On admission, physical examination revealed a girl with a weight of 19 kg (standard deviation score (SDS) = −3.7), a height of 125 cm (SDS = −2.6), head circumference of 57 cm (within normal range for age and gender). No abnormalities in vital signs were detected. She was conscious and generally cooperative during the examination. Other remarkable findings were triangular face, torticollis and facial asymmetry (Figure 1), leg length discrepancy, and right hemihypertrophy. Chewing was disturbed due to right facial hemi hypertrophy. Kyphoscoliosis and asymmetrical lower extremities were also observed (Figures 2, 3, and 4). Parents' examination about syndromic face or posture and torticollis was not significant. There was a normal range of movement in all the extremities, but mild hypotonia, especially of the legs, was observed. In accordance to Tannerstaging sexual maturityscore, she was considered B3P2.

 Neurologic examinations, including cerebellar tests, were unremarkable. Psychiatric tests were also not significant except that she seemed shy with a low self-confidence. 

 Laboratory examination revealed a normal complete blood cell count, biochemistry, liver and thyroid function tests, and venous blood gas analysis. Rheumatologic and metabolic investigation of serum, cerebrospinal fluid, and urine samples showed no remarkable abnormality.

 Neuroimaging and neuromuscular studies were performed; brain MRI, MRV, MRA, and cervical MRI, in addition to electromyography and nerve conduction velocity, produced no positive yields. Ophthalmologic exam by pediatric ophthalmologist showed no retinal changes in favor of hereditary neurometabolic or neurodegenerative diseases. Also no refractory error was found and a compensatory torticollis of ocular origin was ruled out. Sonographic evaluation of neck structures was performed, and a remarkable size difference of sternocleidomastoid (SCM) muscles was revealed (right SCM = 11 mm, left SCM = 6 mm).

 Considering the positive history of IUGR and postnatal growth retardation and fulfilling the phenotypically features of triangular face, short stature, normal head growth, hemi hypertrophy, gradual regression in speech, and after performing a thorough exclusive investigation, she was diagnosed as Russell-Silver syndrome (RSS).

## 3. Discussion

 RSS is a clinically and genetically heterogeneous disorder. It was independently described by Silver et al. [5] who emphasized the short stature and “congenital hemi hypertrophy” of these children and by Russell [6] who focused his report on the “intrauterine dwarfism” and the “craniofacial dysostosis” associated with this syndrome. RSS is considered as an imprinting syndrome. Intrauterine and postnatal growth retardation plus variable additional features including fifth-finger clinodactyly, limb hemi-hyperplasia, a typical facial phenotype, and some learning disabilities characterize it. Despite the recent advances in the genetic characterization, the diagnosis of this syndrome still relies mainly on the clinical phenotype. As compiled from information included in the studies of Netchine et al. [3], Eggermann et al. [7], Bartholdi et al. [8], and Wakeling et al. [9], the diagnosis of RSS and supportive laboratory testing should be considered in individuals who have three major criteria or two major and two minor criteria (Table 1). Nevertheless, when doubtful about the diagnosis in a specific case based on clinical findings, genetic tests may be used to confirm the diagnosis.

The most common genetic mechanism underlying RSS, responsible for approximately half of all cases, is hypomethylation on the paternal allele of H19/IGF2, located at 11p15 [10]. 

 Our patient fulfilled all of the four major criteria, plus one minor criterion (triangular face) and two of the supportive criteria (slurred speech and learning disabilities); so the diagnosis of RSS was clinically certain. But, torticollis, as one of our patient's chief complaints, has not been considered as a classic or even supportive criteria in RSS [7–9].

 Nontraumatic, acquired torticollis has been reported in rare syndromes. Grisel'ssyndrome is a non-traumatic, fixed rotary subluxation of C1 on C2 (atlantoaxial). This syndrome usually is a postoperative complication and can result in acquired torticollis, as reported by Battiata and Pazos [11].

During the course of systemic vasculitis different types of eye involvement and subsequent compensatory torticollis may develop. Churg-Strauss syndrome (allergic granulomatous angiitis) is one of these vasculitides [12]; but in thorough investigation no finding was detected in favor of vasculitis syndromes or eye disease in our patient.

 Severe craniofacial anomalies in RSS are generally uncommon. Some individuals have Pierre Robin sequence and cleft palate. Wakeling et al. [9] found cleft palate or bifid uvula in 7% of their patients with 11p15.5 methylation defects and in no patients with maternal UPD7. Dental and oral abnormalities are rare. Microdontia, high-arched palate, and dental crowding secondary to the relative micrognathia and small mouth have been reported [9, 13–15]. RSS is a constellation of variable and sometimes new manifestations. As said earlier, ultrasonography showed that in our patient asymmetry of SCM muscles resulted in torticollis. As said in case presentation, our patient had a history of IUGR. There exist a few genetic conditions in which IUGR can also be a significant feature including Three M syndrome (3-M), Dubowitz syndrome [16] but in according to major features of these topics, our patient does not fulfill their criteria. 

In conclusion, we are going to emphasize that asymmetry of the limbs is a well-known phenomenon in RSS but asymmetry of SCMs is a remarkable and novel finding.

## Figures and Tables

**Table 1:** Clinical criterion for Russell-Silver Syndrome.

Major criteria	(i) Intrauterine growth retardation/small for gestational age (<10th percentile)
	(ii) Postnatal growth with height/length <3rd percentile
	(iii) Normal head circumference (3rd–97th percentile)
	(iv) Limb, body, and/or facial asymmetry

Minor criteria	(i) Short (arm) span with normal upper-to-lower segment ratio
	(ii) Fifth-finger clinodactyly
	(iii) Triangular faces
	(iv) Frontal bossing/prominent forehead

Supportive criteria	(i) Café au lait spots or skin pigmentary changes
	(ii) Genitourinary anomalies (cryptorchidism, hypospadias)
	(iii) Motor, speech, and/or cognitive delays
	(iv) Feeding disorder
	(v) Hypoglycemia
